# Is prevention of suicide worth less? A comparison of the value per statistical life

**DOI:** 10.1007/s10198-021-01361-6

**Published:** 2021-08-21

**Authors:** Vimefall Elin, Persson Mattias, Olofsson Sara, Hultkrantz Lars

**Affiliations:** 1 School of Business, Örebro University, Örebro, Sweden; 2 Swedish Institute for Health Economics, Lund, Sweden

**Keywords:** Value of statistical life, Willingness to pay, Mental health, Cost–benefit, Altruism, D61, I18, J17

## Abstract

This paper compares the value per statistical life (VSL) in the context of suicide prevention to that of prevention of traffic fatalities. We conducted a contingent valuation survey with questions on willingness to pay (WTP) in both contexts by administering a web questionnaire to 1038 individuals aged 18 to 80. We conjectured that WTP for a given impact on the number of fatalities would be lower for suicide prevention because suicide, at least to some degree, is the result of individuals’ own decisions. However, this hypothesis was not supported by the within- or between-sample estimates of WTP or by responses to direct questions. Hence, no support is provided for the use of a lower valuation of the impact of suicide prevention than for risk-reducing programs in other fields, such as traffic safety. This implies that the same VSL should be used for evaluating suicide prevention interventions and for risk-reducing programs in other policy areas and funds for the prevention of fatalities should be directed to the area with the lowest cost per life saved.

## Introduction

Each year, more than 0.8 million individuals worldwide commit suicide, and several more make suicide attempts [1]. In addition to being a source of tremendous grief among friends and relatives of the victim, suicides give rise to large costs for society [2–4]. The World Health Organization (WHO) member states have committed to working toward the goal of reducing the suicide rate by ten percent by 2020 [5]. However, prevention programs are costly, and tradeoffs must be made with alternative expenditure items, such as the reduction of traffic accidents. The aim of this study is to compare the value of suicide prevention to the value of preventing fatalities caused by other accidents, such as traffic accidents.

One of the core parameters in economic evaluations of programs that affect the risk of premature fatalities is the Value of a Statistical Life (VSL). The VSL is used in cost–benefit assessments of investments and programs within a wide range of policy areas, such as traffic, health, environment and social work. 1Until recently, economic evaluations of suicide prevention programs have been uncommon and have been based on VSL levels derived in another policy context [11]. However, if respondents believe that an individual is the best one to decide if her life is worth living or not and should have the possibility to make that decision, i.e. if a suicidal individual is viewed as a rational decision-maker, this might lower the willingness to pay for suicide prevention. Thereby it could be that individuals are unwilling to spend as many resources per life saved through suicide prevention programs as for programs that target premature non-voluntary fatalities. Therefore, one may argue that VSL for suicide should be lower than for other causes of premature death.

To our knowledge, this is the first study to compare WTP for the prevention of suicide and traffic fatalities based on data collected within the same survey. It is also the first study to estimate WTP for suicide prevention outside of Japan.

We conducted a contingent valuation study with a web questionnaire among a representative web panel of Swedish residents aged 18–80. A total of 1038 subjects were asked to state their willingness-to-pay (WTP) for interventions that were expected to save 100 (200) lives by preventing traffic fatalities or suicides, respectively. They were also asked whether they thought it was more important to reduce the number of deaths due to traffic accidents or due to suicides. Approximately 69 percent stated that these purposes are equally important, while 17 percent stated that suicide prevention is more important, and 13 percent stated the opposite. No support was found for the hypothesis that the WTP for suicide prevention is lower than the WTP for risk-reducing programs related to traffic safety. With regard to policy, this finding suggests that funds for the prevention of fatalities should be directed to the program with the lowest cost per life saved.

The paper is organized as follows. In Background, we review relevant theory, previous literature and the Swedish policy context. Modeling approach describes the study design, questionnaire, empirical strategy and the sample. The results are presented in Results, and in Discussion and conclusion, the paper ends with a discussion and conclusion.

## Background

### Economic theory

In a society with purely self-interested individuals, the VSL is determined by the marginal rate of substitution between own wealth and own risk [12]. The same holds for the valuation of a private good that only has effects on individual risk. However, when the good being valued is public and the individual has preferences for the wellbeing of others, the VSL can also incorporate altruistic preferences. These preferences can further be divided into pure altruism and paternalistic altruism. In the case of pure altruism, the individual cares about the wellbeing of others and respects their preferences; that is, the utility of individual *i* depends on the *utility* of individual *j*. In the case of paternalistic altruism, the individual cares about the wellbeing of others but does not believe that these individuals are the best judges of their own utility; that is, the utility of individual *i* depends instead on some argument in the utility function of individual *j*. If individual *i,* for example, only cares about the safety of individual *j*, individual *i* is said to have safety-focused altruism [13]. The form of such possible altruistic preferences will affect the WTP for suicide prevention because a positive WTP means that the respondent is willing to pay to prevent another individual from taking a certain action.

In their seminal paper on the economics of suicide, Hamermesh and Soss (1974) argue that a utility-maximizing individual commits suicide if the present value of his expected lifetime utility becomes zero [14]. A subject that has purely altruistic preferences can then be expected to have a zero WTP for preventing other individuals from committing suicide. 2 Similarly, if the subject respects the preferences of her future self, the WTP to reduce the own probability of committing suicide would also be zero. If, on the other hand, the subject does not view suicidal-prone individuals as rational decision-makers, the WTP to prevent suicide could be positive, meaning that the subject has paternalistic altruistic preferences. Similarly, if the subject thinks that there is a possibility that her future self is not the best judge of her own utility, then she might be willing to pay to reduce her own risk of committing suicide in the future (i.e., the subject has paternalistic altruistic preference toward herself).

We conjectured that WTP for a given impact on the number of fatalities is lower for suicide prevention if subjects have self-centered or purely altruistic preferences and see suicide as the outcome of a (in some sense) rational decision. Traffic accidents are, as a rule, involuntary (if not caused by a suicide attempt). In contrast, the decision to commit suicide can be seen as either a voluntary decision by a (in some sense) rational individual or as an irrational decision by an individual who is unable to decide what is best for herself. In the former case, subjects with self-centered or purely altruistic preferences have no reason to pay for suicide prevention. In the latter case, people may have paternalistic preferences that result in a positive WTP for suicide prevention that may be lower, equal to or higher than the WTP for the prevention of fatalities caused by traffic accidents.

### Earlier literature

To our knowledge, the only previous literature on WTP for suicide prevention is a series of studies in Japan by Sueki [15–18]. 3 In the first two of these studies, the VSL for suicide prevention was estimated with two different samples (university students and Japanese taxpayers) and different methods (open-ended questions and double-bounded dichotomous choices). This resulted in VSL estimates of USD 0.2 million and USD 0.27 million. These findings were then compared to VSL estimates of approximately USD 2 million for traffic accidents found in other studies in Japan.

Sueki (2016) further investigated how the WTP for suicide prevention is influenced by respondents’ attitudes toward suicide [17]. He found that respondents who think that suicide *can happen to anyone* and that *suicides can be prevented* have, on average, a higher WTP than respondents who state otherwise, while respondents who believe that committing suicide is *an individual right* have a lower average WTP. Furthermore, Sueki (2017) found that WTP can be changed by, for example, giving respondents a series of lectures about suicide before answering the questionnaire [15]. Since attitudes tend to differ depending on the cultural context, it is hard to judge the generalizability of these results. For example, it has been shown that the rate of suicide differs among countries with different religions [19].

Our study differs from these previous studies in several important ways. First, Sueki (2015, 2016a) framed the good as a reduction of *the respondent’s own* probability of dying from suicide [16, 18].^4^

“By implementing the countermeasure, the death risk by suicide for 1 year can be decreased from 20/100,000 to 15/100,000, meaning that your death risk from suicide decreases by 25%.

Imagine national and local governments were to launch the new countermeasure against suicide and collect specific contributions for it.

Do you approve or disapprove of JPY XXX (500; 1000; 2000; 4000; or 8000) tax increase per year to implement the countermeasure against suicide?”.

The task given to respondents in this way is a cognitively complex one because the respondent must think of herself as a current “planner” restricting herself as a future “doer”. If the respondent views herself as a rational utility-maximizing person, the WTP to reduce her own risk of dying from suicide would be zero.

Furthermore, Sueki’s (2015, 2016a) comparison was with VSL estimates in another context (i.e., traffic) from other studies, which means that the results may be confounded by differences in study design [16, 18]. Previous research has shown that the VSL is dependent on context. For example, individuals are often willing to pay a higher premium to reduce the risk of dying from cancer than from other causes [9, 20–22]. 5 Johansson-Stenman and Martinsson (2008) found that respondents reveal a higher valuation of saving pedestrians than car drivers [23]. Similarly, Carlsson et al. (2010a) found that the VSL for fire and drowning accidents seems to be lower than for traffic accidents [24]. One potential explanation to this discussed by the authors is the difference in base line risk between the contexts, with about four times as many deaths due to traffic accidents then due to fire and drowning. However, Carlsson et al. (2010b) found no difference with regard to the cause of an accident [25]. These authors argue that this might be because in the first study, each respondent was asked to value a reduction in her own risk, while in the second study, she was asked to choose between different projects influencing the risk of others.

With regard to issues similar to the subject field of our study, studies show that individuals seem to be willing to pay less for different health care programs targeting mental health compared to both elderly care and cancer programs [26]. Additionally, even though individuals view mental illness as more burdensome than general medical illness, their average WTP is lower for mental health illness [27].

### Swedish policy context

**Fig. 1:**
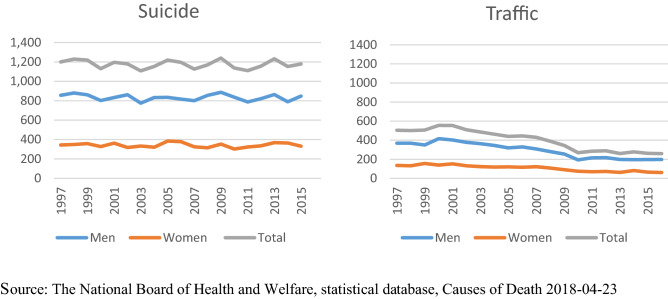
Number of deaths by suicide and traffic.

In 2008, the Swedish Parliament decided on a “vision zero” long-term target for suicides. This policy was inspired by a similar policy from 1997 targeting fatalities and severe injuries caused by traffic accidents. While the policy for traffic has been very successful and has resulted in a decrease in the number of traffic fatalities by close to 50 percent, the number of suicides has been more or less constant since the decision was made (Fig. 1).

In 2016, 1134 individuals committed suicide and 259 died in traffic. More men than women die both in traffic and from suicides. Both suicides and deaths due to traffic accidents are present in all age categories (Table 1).

**Table 1:** Number of deaths 2016, by age, by suicide and traffic

Age	Suicide		Traffic	
	(%)	*n*	(%)	*n*
0–29	17	195	22	56
30–49	29	334	20	52
50–69	33	378	30	78
70–	20	227	28	73
Total	100	1134	100	259

Source: The National Board of Health and Welfare, statistical database, Causes of Death 2018-04-23

Within traffic policy, there is a long tradition of conducting cost–benefit analysis, and the VSL has become a core parameter in this context. Hultkrantz and Svensson (2012) summarized twelve different studies, including 48 VSL estimates, conducted in Sweden between 1996 and 2010. Most of these (39/48) were based on traffic safety, but this review also included studies from other policy contexts, such as health care, air travel, fire and drowning [8]. The current recommendation by the Swedish Transport Administration is to use a VSL of 4.82 million USD^6^ and is partly based on results from Olofsson et al. (2016) [28].

## Modeling approach

### Study design

To estimate the VSL, we conducted a contingent valuation study. Because traffic is the policy area where most previous VSL studies have been performed, estimating the WTP for traffic safety and suicide prevention within the same study can provide important insight into how and why these values might diverge from each other. To further facilitate comparison, our study design and analysis of responses is similar in several respects to the methods used by Olofsson et al. (2019b), which is the most recent Swedish study for the elicitation of VSL in a traffic-safety context [10].

The survey started with an introduction explaining the purpose and providing some practical information. This was followed by background questions on age, gender, marital status, country of birth, number of persons in the household (total and under 18), education, occupation, and life satisfaction. This section ended with a question regarding the respondent’s opinion on the importance of interventions that could save lives with regard to traffic safety and suicide prevention. The survey consisted of two WTP sections, one about traffic and one about suicide. To check for scale sensitivity, both sections included two levels of absolute risk reduction (100 and 200 saved lives). After a short introduction to the WTP concept, both of these sections initially showed information on the number of people who die in traffic/by suicide each year by age and gender. Approximately 80 percent of the respondents were given the questions about traffic before the questions about suicide, while approximately 20 percent received these questions in the opposite order.

The respondents were asked whether they thought the government should spend money on an intervention that would save 100/200 lives within the specific policy context (Appendix A1). Because previous studies have shown that respondents have a difficult time understanding a reduction in small probabilities [28], the risk reduction was presented as the absolute number of lives saved. However, information was also provided about the risk reduction expressed as the change in probability for a random individual. The WTP questions followed a variation of the bidding game method that ended with an open-ended WTP question, similar to the method used in Olofsson et al. (2019b) [10]. Each respondent was asked about a maximum of four different payment levels from a tree structure (Appendix A2). The respondent was subsequently shown the maximum bid for which she answered yes and the minimum bid for which she answered no and was asked to state her maximum WTP within this range. To control for starting point bias, the respondents were divided into three groups starting at different values. The respondents were informed that the payments would be collected through a uniform tax, and they were shown both the total payment by all taxpayers and the cost per taxpayer.

Respondents were further asked to specify how certain they were that in reality they would vote yes to a proposal to run the intervention using a 0–10 Likert scale and whether they would be willing to donate the money they would receive for answering the survey to support interventions to improve traffic safety or to support mental health programs.

To ensure that the final answer was consistent with the individual’s preferences, each respondent was shown a comparison of her stated WTP for saving 100 lives in both contexts and asked whether she wanted to change her answer. Feedback was given by a text stating, for instance, “*this indicates that you think it is worth more to save a life related to traffic than by suicide reduction*” and then asking whether this statement was correct. If the stated WTP for one area was larger than the WTP in another area, the respondent was asked why. Because this may have induced the respondents to state equal WTPs because they thought this was the right thing to do, both the responses to the first open-ended question and the (potentially changed) final answer were used in the analysis.

A final section of the survey consisted of questions regarding each respondent’s own experience with traffic accidents and mental health problems. The respondents were also asked questions regarding their attitude toward mental health problems in general and suicide in particular. Finally, they were asked to evaluate their own quality of life on a Likert scale.

### Pilot study

As a test, the survey was sent to 50 respondents in November 2017. In addition, we conducted a focus group study with five students. The pilot indicated that some respondents did not consider the opportunity cost of public funds. Therefore, in the final survey, a sentence was included describing what the same amount of money could buy in terms of numbers of preschool teachers, doctors, nurses, and police officers. Additionally, the outline was changed, starting with the total societal cost and showing the payment per individual in parentheses. Apart from this, only minor changes were made.

### Empirical strategy

Because the WTP is censored from below at zero, when analyzing the determinants of WTP, a standard Tobit model was estimated.

$$y_{i}^{*} = x_{i}^{^{\prime}} \beta + \varepsilon_{i}$$

$$y_{i} = ~y_{i}^{*} ~\,\,\,\,if\,\,\,\,\,\,~y_{i}^{*} > 0$$

$$y_{i} = 0~\,\,\,\,\,\,if\,\,\,\,\,\,~y_{i}^{*} \le 0,$$

where $y_{i}$ is the stated WTP of individual *i* and $\varepsilon_{i}$ is assumed to be NID(0,σ^2^) and independent of *x*_*i*_.

To analyze the size of the difference in WTP in the two contexts, a variable for this difference (i.e., WTP_suicide_–WTP_traffic_) was generated. This was analyzed using a standard OLS.

The same set of explanatory variables was used in all analyses. In the baseline specification, only age (and age squared), gender and education (any tertiary education or not) were included. The effect of income was analyzed separately because this variable contained many missing values. In a second step, variables capturing the respondent’s experiences and attitudes were included. For suicide, a dummy variable indicated whether the individual knew someone who had tried to commit or had committed suicide. Additionally, a variable indicating whether the respondent had ever sought help for depression was included. Two other variables indicated whether the respondent thought that she had control over her probability of experiencing depression and whether, according to her opinion, it should be an individual’s own decision to end her own life. Other included variables indicated whether the respondent knew anyone who had died from a traffic accident, whether she herself had ever been in a traffic accident, whether she thought that her risk of being in a traffic accident was higher or lower than that of the general population, and finally her concern, on a 1–10 scale, of her own risk of being involved in a traffic accident. Finally, a set of dummy variables was included, indicating how often the respondent traveled by car, as a driver and as a passenger.

### The sample

In total, 3908 individuals were invited to participate in the survey, all of whom were participants in the Norstat panel, a telephone-recruited web panel consisting of 67000 individuals. Of these, 1197 (31 percent) started the survey and 1038 (27 percent) completed the full survey. All participants received a small reward (points that could be exchanged for money), which they could choose to donate to charity. The sample was drawn to be representative of the total population with regard to age groups, gender and region (Table 2). In the sample, approximately 50 percent were married, 23 percent were born in a country other than Sweden, and 26 percent had at least one person under the age of 18 living in the household. The majority, 54 percent, had some form of tertiary education (at least some education from a university). With regard to employment, approximately 52 percent were employed and 32 were retired. Compared to the population, we see that our sample is somewhat older, more likely to be married and to have some higher education. These individuals are also less likely to be employed or self-employed but are more likely to be retired. This shows that our sample is not representative for the general population on other characteristics then what the sample was based on (gender, geography and age groups) and it will be important to control for how these variables influence the result.

**Table 2:** Descriptive statistics, full sample, *n* = 1038, and population

	Sample (%)	Population (%)	Diff
Gender			
Men	50.39	50.55	− 0.16
Women	49.42	49.45	− 0.03
Age (mean)	50.43	47.04	3.39***
Marital status			
Not married	37.57	42.41	− 4.84***
Married/partnership	50.96	42.42	8.54***
Widow/widower	3.95	2.95	1.00*
Divorced	7.51	12.22	− 4.71***
Country of birth			
Sweden	75.82	78.72	− 2.9**
Outside of Sweden	23.41	21.28	2.13
Highest level of education^a^			
Primary education	8.09	16.39	− 8.3***
Some secondary education	38.24	44.28	− 6.04***
Some university or more	53.66	37.00	16.66***
Employment status^b^			
Employed or self-employed	56.64	67.84	− 11.2***
Retired	31.50	12.77	18.73***
Student	6.94	7.05	− 0.11
Searching for job	2.31	5.38	− 3.07***
Other	2.60	6.93	− 4.33***

All population statistics are from 2017 for ages 18–80 (Statistics Sweden Statistical Database)

^a^In the population 2.33%, there is no educational information

^b^For the general population, this considers age group 15–74

Significant at levels ***1%, **5%, *10%

## Results

### Attitude and preferences

Respondents’ answers to the question of which policy area, traffic safety or suicide prevention, was most important are shown in Table 3. Most respondents stated that they perceived both areas as equally important. However, statistically significantly more respondents answered that suicide reduction was more important than reducing the number of deaths due to traffic accidents.

**Table 3:** What do you think is most important?

	Frequency	Percentage
To reduce the number deaths due to traffic accidents	140	13.5
To reduce the number of suicides	181	17.4
I think both policy areas are equally important	717	69.1
Total	1038	100

Regarding previous experience, 19 percent stated that they had been in a traffic accident that required them to go to a hospital and 14 percent knew someone who had died in a traffic accident. 19 percent stated that they were worried that they would be hurt in a traffic accident.^7^

With regard to suicide, 54 percent knew someone who had committed suicide or had tried to do so. Furthermore, 63 percent stated that they thought society should take vigorous action to reduce the number of suicides, 8while 23 percent agreed with the statement that each individual should be allowed to decide whether they would like to end their life.^9^

Among those who stated a higher WTP for suicide prevention than traffic safety (*n* = 180), the main reason was “I think society does too little in this area” (*n* = 89), followed by “I think there are more possibilities to reduce the number of deaths within this area”. Those who stated a higher WTP for traffic than suicide prevention (*n* = 157) stated, “I think it is more important to prevent an involuntary death then a voluntary death” (*n* = 62) and “I think there are more possibilities to reduce the number of deaths within this area” (*n* = 58).

Each respondent received a small monetary reward for answering the survey. When asked whether they would be willing to donate this to support different types of interventions, approximately 54 percent indicated their willingness to do so. 10 Thirteen percent stated that they would be willing to donate the money to mental health programs, 5 percent stated that they would be willing to donate the money to traffic safety programs, 30 percent would be willing to donate to both types of programs, and 7 percent would donate to programs in other fields.

### Willingness to pay

In the WTP section, for each scenario, the respondents were asked to make a maximum of four binary (accept or decline) choices for cost bids that were raised or decreased depending on the previous response. Finally, the respondent received an open-ended question asking about the maximum WTP. In the following analysis, we used the answer from the open-ended question (Table 4).

**Table 4:** Willingness to pay, (USD)

	WTP suicide prevention		WTP traffic safety	
	Mean (standard deviation)	Min; Max	Mean (standard deviation)	Min; Max
Scenario 1: 100 lives				
Full sample (*n* = 1019)	289.7 (3736.9)	0; 119,067.5	6 708.4 (187,438.3)	0; 5,953,373.2
Without outliers (*n* = 1014)	172.4 (304.2)	0; 3752	164.9 (295.9)	0; 3752
Without irrational (*n* = 750)	175.6 (312.3)	0; 3752	159.5 (297.4)	0; 3752
Without uncertain responses (*n* = 675)	175 (315.7)	0; 3752	157.9 (296.2)	0; 3752
Scenario 2: 200 lives				
Full sample (*n* = 1019)	1339.7 (37,295.1)	0; 119,067.5	6299.7 (186,552.5)	0; 5,953,373.2
Without outliers (*n* = 1014)	230.8 (446.3)	0; 4762,7	222.9 (420.8)	0; 4762.7
Without irrational (*n* = 750)	262.1 (475.7)	0; 4762,7	252.3 (459.7)	0; 4762.7
Without uncertain responses (*n* = 675)	258.1 (475.4)	0; 4762,7	246.3 (454.6)	0; 4762.7

Using the uncleaned data, we find a mean WTP of 289.7 USD for suicide and 6708.4 USD for traffic. Even though this difference is large, it can be explained by some extreme outliers where one individual stated a WTP of 5,953,373 USD and one a WTP of 595,337 USD, which for both individuals are high above their yearly household income and thereby can be viewed as unrealistic. Cleaning the data from such extreme outliers will, of course, have a large effect on the mean WTP but does produce more reliable results.^11^

**Fig. 2:**
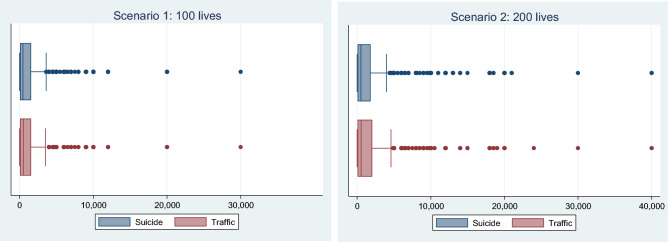
Willingness to pay to save 100 and 200 lives

After cleaning the data from extreme outlier, we did not find support for the hypothesis that the WTP for suicide prevention is lower than the WTP to reduce the number of deaths within traffic. On the contrary, the results suggest a somewhat higher WTP for suicide prevention. However, using a Wilcoxon signed-rank test, we found that the difference was only statistically significant in the scenario with 100 lives after we dropped the irrational respondents, i.e., those who stated a higher WTP for the lower risk reduction. The distribution of the final WTP responses for both suicide and traffic is presented in Fig. 2.^12^

**Fig. 3:**
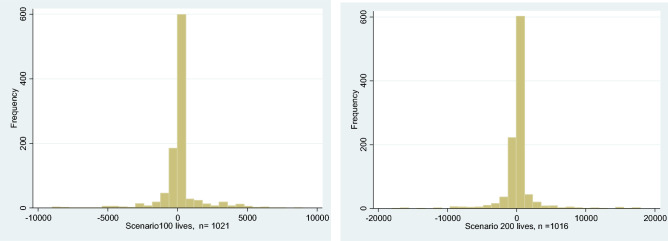
WTP suicide–WTP traffic

Figure 3 shows the difference between the WTP to save 100 lives through a reduction in the number of suicides and the WTP to save 100 lives through a reduction in the number of deaths from traffic accidents.

The null hypothesis that the WTP for suicide prevention is larger than or equal to the WTP for traffic safety could not be rejected in a t-est when using the full sample. As a robustness test, the test was re-run step by step for 100 and 200 lives including all respondents and then excluding outliers, 13 respondents who stated a higher WTP to save 100 lives than to save 200 lives, and those who were uncertain^14^ (Table 5).

**Table 5:** Test H_0_ = mean (WTPs–WTPt) ≥ 0 (*p* value in parenthesis)

	Scenario 1: 100 lives	Scenario 2: 200 lives
Full sample (*n* = 1019)	Cannot reject H_0_ (0.137)	Cannot reject H_0_ (0.205)
Without outliers (*n* = 1014)	Cannot reject H_0_ (0.907)	Cannot reject H_0_ (0.801)
Without irrational (*n* = 750)	Cannot reject H_0_ (0.996)	Cannot reject H_0_ (0.821)
Without uncertain responses (*n* = 675)	Cannot reject H_0_ (0.998)	Cannot reject H_0_ (0.865)

In none of these specifications was support found for the hypothesis that the average WTP from suicide prevention is lower than the average WTP for saving a life in traffic. On the contrary, when using the lower risk reduction (100 lives) and after cleaning the data for outliers, using a two-tailed test, we found that the average WTP for suicide prevention is statistically significantly higher than the average WTP for preventing fatal traffic accidents (Appendix A3). 

A further set of tests was conducted using the WTP values stated before the respondents were able to change their answers. 15 In these tests, no support was found for the hypothesis that the average WTP for suicide prevention is lower than the average WTP for saving a life within traffic. Furthermore, a test was conducted regarding the difference between the responses of those who started with different scenarios using only the first scenario, which showed no significance.

### VSL

To make a possible comparison with other studies, the VSL was computed as in Eq. 1 based on the assumption that there are 8,000,000 taxpayers in Sweden.^16^

1 $$VSL_{s} = \frac{{WTP_{s} }}{{\Delta deaths_{s} }} \times 8 ,000, 000$$

where Δdeaths is 100 in *s* = 1 and 200 in s = 2.

To further facilitate comparison, the VSL was computed with results from our study that were cleaned of unrealistic answers following the same procedure for cleaning the data as in Olofsson et al. (2016) [28]. Responses from respondents who stated a higher WTP to save 100 lives than to save 200 lives (269 respondents) were dropped, while those who stated the same amount for both scenarios were retained. The data were also cleaned of responses from protesters and outliers. 17 Untrimmed answers are provided in the appendix (A4). Descriptive statistics for the trimmed answers are presented in Table 6.

**Table 6:** Median, mean and standard deviation for VSL. (USD)

	Suicide *n* = 745, *n* = 738		Traffic *n* = 760, *n* = 737	
	Median	Mean (standard deviation)	Median	Mean (standard deviation)
Δdeaths = 100	2,381,349	6,096,254(8,013,240)	2,643,298	6,096,254 (7 644,131)
Δdeaths = 200	1,905,079	4,107,828 (5,215,155)	2,381,349	4,203,082 (4,893,673)

The estimated VSL reported in Table 6 is in line with the result by Olofsson et al. (2016) [28] and can be compared to the latest recommendation by the Swedish Transport Administration, which is 4.82 million USD. As expected, the VSL is lower when using the larger risk reduction.

### Determinants of WTP

Women seem to have a higher WTP for suicide prevention than men, and respondents with higher education have a lower WTP for both scenarios (Table 7). Apart from this, the attitude toward mental health in general and suicide in particular seems to be important. Those who think that it is possible to control their own risk of experiencing depression have a lower WTP for suicide prevention. The same holds true for individuals who state that they think it should be up to an individual to end her own life. This finding is in line with Sueki (2016b) [17], who showed that respondents’ attitudes toward suicide are important for their WTP. This shows the importance of attitudes, which might differ due to different cultural contexts. In the case of traffic safety, most coefficients have the expected sign, but only a few are statistically significant.

**Table 7:** Determinants of WTP, Tobit regression

	WTP suicide	WTP traffic	WTP suicide	WTP traffic
Constant	38.03 (86.68)	39.57 (83.57)	70.45 (87.80)	33.84 (101.76)
Age	5.14 (3.68)	5.00 (3.55)	5.18 (3.68)	5.26 (3.65)
Age^2^	− 0.05 (0.03)	− 0.05 (0.03)	− 0.05(004)	− 0.05 (0.04)
Women	67.42 (20.40)***	15.20 (19.62)	62.72 (20.90)**	14.28 (21.16)
Higher education	− 40.93 (20.45)**	− 34.54 (19.66)*	− 42.58 (20.47)**	− 34.73 (20.00)*
Experience of suicide			11.41 (20.74)	
Depression			− 0.69 (24.91)	
Control			− 43.57 (21.91)**	
Attitude—own choice			− 47.70 (22.92)**	
Traffic accident/death				23.86 (27.96)
Experience own traffic accident				− 5.58 (25.21)
Worry traffic risk				2.01 (4.20)
Subjective risk				− 0.33 (5.64)
*N*	1023	1023	1023	1023
Censored observations	91	72	91	69
Pseudo *R*^2^	0.0010	0.0003	0.0015	0.0013

The second specification about WTP for traffic also includes dummy variables capturing how often the respondent traveled by car as a driver or as a passenger; none of these were statistically significant

Standard errors in parentheses. ***p < 0.01, **p < 0.05, *p < 0.1

Controlling for the size of the first bid shows that individuals who started with the highest bid had, on average, a statistically significant higher WTP than respondents who started with the lowest bid^18^. Dropping irrational respondents makes the coefficient for age statistically significant in all specifications. Income is not statistically significant.

These results are important for the generalizability of our conclusions. For example, we find that individuals with higher education have a lower WTP both for traffic safety and the reduction of number of suicides. This is important since this group is overrepresented in our sample compared to the population in general, which could introduce a positive bias in our estimations.

However, analyzing the size of the difference (wtp_suicide_–wtpt_traffic_), the only statistically significant results are that the coefficient for women has a positive effect and the belief that individuals should have the right to decide when to end their own life has a negative effect.^19^ This indicates that even if our sample is not representative in respect to education this does not seem to influence the conclusion regarding the difference between contexts.

Furthermore, these results point at the importance of considering attitudes and cultural factors when analyzing the WTP for suicide prevention. This is important when comparing our results with studies from other countries which might differ in their attitudes to suicide.

## Discussion and conclusion

In this paper, we conducted a contingent valuation study to compare the WTP for suicide prevention with the WTP to save a life from a traffic accident. To our knowledge, this is the first paper to estimate these two values in the same survey and the first to estimate the WTP for suicide prevention outside of Japan. Our hypothesis was that the WTP for a given impact on the number of fatalities for suicide would be lower than the WTP to reduce the number of fatalities from traffic accidents because suicides are, to some degree, the result of individuals’ own decisions. However, contrary to our hypothesis, we did not find that individuals have a lower WTP for a suicide prevention program compared to a traffic safety program with equal reduction of the number of fatalities.

One interpretation of our results is that the WTP responses reveal paternalistic altruism, i.e., individuals are willing to pay to change the behavior of others and/or their future selves. A reason for this could be that many individuals do not believe suicide to be a rational decision. This may be, for example, because many suicides are connected to mental illness and/or substance abuse, situations in which the individual is not viewed as a rational decision-maker.

An aspect that could have an influence on the WTP is the fact that in Sweden, unlike the situation on the global scale, more people die by suicide than in traffic accidents. The respondents were informed about the number of deaths in the two cases, which may have influenced their answers. This might also influence the generalizability of the results.

We are aware that the contingent valuation method has several problems that could affect the validity of our results. Although we attempted to control for some of these issues, they might bias the VSL estimates. However, our main goal was to study whether there is a difference in WTP for suicide prevention compared to traffic safety. Because both values were estimated in the same survey, this bias can be expected to go in the same direction. Additionally, we used a similar survey instrument and analyzed the results in a similar way as in the most recent traffic safety VSL study in Sweden.

Our results diverge from those of previous studies conducted in Japan by Sueki (2015, 2016a) [16, 18], which indicated that the VSL in the context of suicide prevention is lower than for traffic accidents. However, since these studies compare the VSL from different studies, it is hard to know if their result is due to a real difference in valuation or if it is an artifact due to methodological differences between the studies. This is extra important when it comes to stated preferences studies since the derived VSL often varies a lot. For example, Hultkrantz and Svensson (2012) [8] report results from published WTP studies of the VSL for traffic safety in Sweden that vary between 9 and 153 MSEK. Thereby, to be able to draw conclusions regarding the importance of context, it is important to do this within the same study, keeping the method constant.

The main methodological differences between our study and that of Sueki (2016a) [16] are that Sueki asks about the individuals WTP to reduce her *own* risk of dying from suicide in the future. This means that if the respondent has a positive WTP, she is willing to pay to hinder herself from taking a specific action in the future, i.e., she has altruistic paternalistic preference for her future self.

An additional difference is of course the country context in which the studies are conducted and potential differences in attitudes to suicide. This is important because if the individual views suicide as a rational decision, then, based on the theoretical argument, the WTP for suicide prevention would be lower. The importance of attitudes is also supported by the empirical evidence both from our study and in Sueki (2016b) [17]. For example, we find that respondents who state that individuals should have the right to decide when to end their own life have on average a statistically significant lower WTP for suicide prevention compared to others.

With regard to policy conclusions, our results do not find support for the hypothesis that the average WTP is lower for the prevention of suicide than for life-saving interventions in other policy areas, such as traffic. This implies that the same VSL should be used for evaluating suicide prevention interventions and for risk-reducing programs in other policy areas. Funds for the prevention of fatalities should be directed to the area with the lowest cost per life saved.

As noted above, the national “vision zero” program from 2008 has not been very successful. To get more energy in this kind of work, the Swedish government recently commissioned two government agencies to provide input for the design of a new national strategy to be launched in 2023/2024. These preparations will be pursued in collaboration with twenty other government agencies, among them some, such as the National Transport Administration, that regularly base priorities on benefit–cost assessments. The government’s goal is to strengthen control, enhance coordination among government agencies in various policy fields and on national, regional and local levels, and improve overall quality, efficiency and persistence of efforts to prevent suicides and promote public mental health. We hope that results from studies like this one can provide useful pieces of information in the design of such strategies.

## Data Availability

The data are not publicly available but might be sent to external reviewer after ethical approval.

## Appendix A1

Example from the questionnaire (numbers in SEK, 1SEK ≈ 0,12USD)

Do you think that the intervention should be done?

Yes No.

## Appendix A2

Structure of the cost bids.

Numbers in SEK, 1SEK ≈ 0,12 USD.

Scenario 1: 100 lives.

Scenario 2: 200 lives.

## Appendix A3

See Table 6.

**Table:** Two-tailed test

	Scenario 1: 100 lives		Scenario 2: 200 lives	
	Mean (Std dev)	*T* test (*p* value)	Mean (Std dev)	*T* test (*p* value)
Full sample (*n* = 1019)	− 6419 (187,408)	Cannot reject H_0_ (0.275)	− 4900 (189,555)	Cannot reject H_0_ (0.4095)
Without outliers (*n* = 1014)	8 (182)	Cannot reject H_0_ (0.1869)	8 (301)	Cannot reject H_0_ (0.3985)
Without irrational (*n *= 750)	16 (164)	Can reject H_0_ (0.0076)	10 (291)	Cannot reject H_0_ (0.3591)
Without uncertain responses (*n* = 675)	17 (156)	Can reject H_0_ (0.0044)	12 (278)	Cannot reject H_0_ (0.2691)

H_0_ = mean (WTPs–WTPt) = 0

**Table:** VSL uncleaned and cleaned answers

	Suicide		Traffic	
	Median	Mean (std)	Median	Mean (std)
Specification 1: full sample				
Δdeaths = 100 (*n* = 1029, *n* = 1027)	4,286,429	23,218,155 (297,668,659)	4,762,699	532,231,562 (14,883,432,953)
Δdeaths = 200 (*n* = 1025, *n* = 1025)	2,381,349	54,651,966 (1,488,343,295)	2,619,484	251,232,348 (7,441,716,477)
Specificatio*n* 2: without outliers (WTP > 10,716)				
Δdeaths = 100 (*n* = 1028, *n* = 1024)	4,262,615	13,930,893 (24,408,830)	4,762,699	13,335,556 (23,694,425)
Δdeaths = 200 (*n* = 1024, *n* = 1021)	2,381,349	9,334,889 (17,860,120)	2,524,230	9,049,127 (17,145,715)
Specificatio*n* 3: without irratio*n*al (WTP 100 lives < WTP 200 lives)				
Δdeaths = 100 (*n* = 866, *n* = 854)	3,810,159	14,049,961 (25,242,302)	3,810,159	12,383,016 (22,980,020)
Δdeaths = 200 (*n* = 862, *n* = 848)	2,857,619	10,489,844 (19,050,794)	3,333,889	9,823,066 (17,860,120)
Specificatio*n* 4: without protesters				
Δdeaths = 100 (*n* = 860, *n* = 850)	3,810,159	14,049,961,(25,242,302)	3,810,159	12,383,016 (22,980,020)
Δdeaths = 200 (*n* = 860, *n* = 845)	2,857,619	10,513,657 (19,169,862)	3,572,024	9,858,786 (17,860,120)
Specificatio*n* 5: without outliers (WTP > 6 x Q3-Q1))				
Δdeaths = 100 (*n* = 844, *n* = 836)	3,810,159	11,716,238 (17,979,187)	3,810,159	10,525,564 (16,312,243)
Δdeaths = 200 (*n* = 839, *n* = 827)	2,381,349	8,406,163 (13,216,488)	3,048,127	8,048,961 (12,502,084)
Specificatio*n* 6: without outliers ( WTP > 1.5x Q3-Q1))				
Δdeaths = 100 (*n* = 747, *n* = 760)	2,381,349	6,096,254 (8,013,240)	2,643,298	6,096,254 (7,644,131)
Δdeaths = 200 (*n* = 738, *n* = 737)	1,905,079	4,107,827 (5,215,155)	2,381,349	4,203,081 (4,893,673)

## Appendix A4

Cleaning for VSL suicide, scenario 100 deaths: from specifications 1 to 2, we dropped 1 respondent who stated a WTP of 11,907 (VSL = 952,560,000). From specifications 2 to 3, we dropped 158 respondents, all of whom stated a higher WTP to save 200 lives than 100 lives. Respondents who stated the same amount for 100 and 200 lives were retained since this can be explained by budget constraints. From specification 6, observations were dropped; however, this did not influence the result. In the last steps trimming for outliers we followed Olofsson et al. (2016) [28]. This step was done to be able to compare our results with previous studies that have followed this approach.

In the scenario with 200 deaths, we found a large drop in the VSL from specifications 1 to 2. In this step, we dropped only one observation. However, this respondent stated a WTP of 1 190 675 (VSL = 47,627,000,000).

For traffic, we also observed a large drop in the in VSL from specifications 1 to 2. In the scenario with 100 lives, we dropped three respondents in this step who stated WTP 119 067 464 (VSL = 9,525,397,120,000), 595 337 (VSL = 47,626,960,000) and 5,953,373 (VSL = 476,269,840,000).

## Footnotes

1 For reviews of previous studies, see de Blaeij et al. (2003) [6], OECD (2012) [7]. Some Swedish VSL studies are reviewed in Hultkrantz and Svensson (2012) [8]. More recent Swedish VSL studies are Olofsson et al. (2019a and 2019b) [9, 10].

2 However, even in this case, people may have a positive WTP for the prevention of suicide considering the grief and other harm to the next of kin that suicide evokes.

3 The Sueki 2015 paper [18] is only available in Japanese; therefore, all information regarding this study is gathered from the Sueki 2016a paper [16].

4 However, it is stated that it is a public intervention.

5 However, whether there should be a cancer premium remains an ongoing research question. For example, Hammit and Haninger 2010 do not find a statistically significant higher WTP for cancer compared to other contexts [29].

6 All Swedish values in this study have been changed to USD using the exchange rate of 8,3986SEK/UDS which was the average exchange rate the month when this study was conducted.

7 Defined as answering higher than six on the question, *How worried are you that you will be hurt in a traffic accident?* (scale 0–10).

8 Defined as answering higher than six on the statement,”*I think society should take vigorous actions to reduce the number of suicides*” (scale 0–10).

9 Defined as answering higher than six on the statement, “*Each individual should get to decide if he/she would like to end his/her own life; this is no one else’s business*” (scale 0–10).

10 This was a hypothetical question, and no actual donation was made.

11 Of these five, one stated a WTP for saving 100/200 lives in traffic of 5 953 373 USD; one stated a WTP for saving 100 lives in traffic of 595 337 USD while only stating a WTP to save 200 lives in the same context of 595 USD; one stated a WTP of 119 067 USD in every scenario/context; one stated a WTP to save 200 lives within traffic of 13 097.4 USD while stating a WTP of only 309.5 USD to save 100 lives; and the last respondent stated a WTP for saving 200 lives within traffic of 13 097.4 USD (only 309.5 USD for 100 lives).

12 Five outliers were excluded; 4 stated a WTP > 95 254 USD and 1 stated a WTP > 11 907 USD.

13 Same as note 8.

14 Respondents were defined as uncertain if they answered < 5 on the question regarding how certain they were that they actually would accept the proposed tax increase.

15 Results available upon request.

16 This assumption was also made in the questionnaire when showing the total cost for society.

17 A outlier was defined as an individual stating a WTP > *1,5* × *the interquartile distance (Q3-Q1).*

18 Results avaible upon request.

19 Results avaible upon request.

## Code availability

Code available upon request.
